# Phthalimide Derivative Shows Anti-angiogenic Activity in a 3D Microfluidic Model and No Teratogenicity in Zebrafish Embryos

**DOI:** 10.3389/fphar.2019.00349

**Published:** 2019-04-17

**Authors:** Annalisa Mercurio, Lucy Sharples, Filomena Corbo, Carlo Franchini, Angelo Vacca, Alessia Catalano, Alessia Carocci, Roger D. Kamm, Andrea Pavesi, Giulia Adriani

**Affiliations:** ^1^Department of Pharmacy-Drug Sciences, University of Bari Aldo Moro, Bari, Italy; ^2^BioSystems and Micromechanics IRG, Singapore-MIT Alliance for Research and Technology, Singapore, Singapore; ^3^Sheffield Institute of Translational Neuroscience, The University of Sheffield, Sheffield, United Kingdom; ^4^Institute of Molecular and Cell Biology, Agency for Science, Technology and Research, Singapore, Singapore; ^5^Department of Biomedical Sciences and Human Oncology, University of Bari Aldo Moro, Bari, Italy; ^6^Department of Biological Engineering, Massachusetts Institute of Technology, Cambridge, MA, United States; ^7^Singapore Immunology Network, Agency for Science, Technology and Research, Singapore, Singapore

**Keywords:** angiogenesis, 3D microfluidics, phthalimide derivative, Thalidomide, zebrafish embryo, teratogenicity

## Abstract

Angiogenesis is a crucial event for tumor progression and metastasis. It is the process through which new blood vessels are formed and has become a therapeutic target in many cancer therapies. However, current anti-angiogenic drugs such as Thalidomide still have detrimental teratogenic effects. This property could be caused by the presence of chiral carbons, intrinsic to such compounds. We synthesized four different *phthalimide* derivatives that lack chiral carbons in their chemical structure. We hypothesized that these achiral carbon compounds would retain similar levels of anti-angiogenic activity whilst reducing teratogenic effects. We tested for their anti-angiogenic functions using an *in vitro* 3D microfluidic assay with human endothelial cells. All four compounds caused a drastic inhibition of angiogenesis at lower effective concentrations compared to Thalidomide. Quantification of the blood vessel sprouting in each condition allowed us to classify compounds depending on their anti-angiogenic capabilities. The most effective identified compound (C4), was tested *in vivo* on a zebrafish embryo model. Blood vessel development was measured using number and lengths of the stalks visible in the *fli1a*:EGFP transgenic line. Potential teratogenic effects of C4 were monitored over zebrafish embryonic development. The *in vivo* results confirmed the increased potency of C4 compared to Thalidomide demonstrated by results in embryos exposed to concentrations as low as 0.02 μM. The teratogenic analysis further validated the advantages of using C4 over Thalidomide in zebrafish embryos. This study highlights how the use of *in vitro* 3D model can allow rapid screening and selection of new and safer drugs.

## Introduction

Angiogenesis is a complex and finely regulated process that consists of the growth of new capillary blood vessels from pre-existing vessels to create communication pathways among tissues. The coordination of different activities such as endothelial cell proliferation and migration, metalloproteinase function, integrin expression and pericyte stabilization is essential for angiogenesis (Folkman, 1984). The “angiogenic switch” is “on” when the balance between pro-angiogenic and anti-angiogenic molecules is disrupted in favor of angiogenesis. A variety of physiological processes rely on angiogenesis such as embryogenesis, female reproductive cycle, organ differentiation and wound healing (Duran et al., 2011). However, angiogenesis also plays an important role in many diseases such as ischemic diseases, vascular malformations, inflammatory disorders, obesity and tumors (Folkman, 1971; Carmeliet and Jain, 2000). In particular, angiogenesis plays a vital role in invasive tumor growth and metastasis and represents an essential mechanism to target and control cancer progression. Angiogenesis allows the recruitment of new blood vessels that become the principal route through which the tumor cells enter the circulation, after leaving the primary tumor site (Zetter, 1998). In addition, the new vessels promote primary tumor growth. Conversely, tumor masses lacking infiltrated capillaries become dormant and are unable to growth beyond 4 mm (Folkman and Hochberg, 1973). This limiting step indicates that blocking pathological angiogenesis may be an effective therapeutic strategy (Jain, 2005).

It was recently observed in patients with active multiple myeloma (MM), that Thalidomide treatment causes a significant reduction in the expression of angiogenic factors such as vascular endothelial growth factor (VEGF), fibroblast growth factor (FGF), hepatocyte growth factor (HGF), insulin-like growth factor 1 (IGF-1), angiopoietins 1 (Ang-1) and 2 (Ang-2) (Vacca et al., 2005).

The Food and Drug Administration (FDA) approved Thalidomide for the treatment of Erythema Nodosum Leprosum (ENL) in 1998 and of Multiple Myeloma (MM) in 2006 because the compound was shown to inhibit angiogenesis through VEGF downregulation (Kenyon et al., 1997; Komorowski et al., 2006). However, Thalidomide administration has been correlated to many adverse effects such as asthenia, rashes, constipation, somnolence, neuropathy, leucopenia and hepatotoxicity (Clark et al., 2001; Chaudhry et al., 2002; Hanje et al., 2006) and deep vein thrombosis (DVT; Minnema et al., 2003; García-Sanz, 2006).

Thalidomide’s devastating teratogenic effects were observed in more than 100,000 children when Thalidomide was first marketed for the treatment of “morning sickness” during pregnancy. These side effects are considered intrinsic to the drug’s chemical structure because of the presence of a chiral carbon that generates two different enantiomers (R and S) that interconvert (Nguyen et al., 2006). Researchers have therefore been addressing the teratogenicity of Thalidomide by studying its precise molecular mechanism (Ito et al., 2010) as well as developing alternative compounds (Bartlett et al., 2004).

Many analogs of Thalidomide have been synthesized and tested such as Lenalidomide and Pomalidomide, developed by CELGENE for the treatment of blood cancer and immune-disorders (Hideshima et al., 2015). However, these compounds retain a chiral carbon, and have a limited use in treating MM patients as they were found to trigger central neurotoxicity (Patel et al., 2015). To overcome these adverse effects, we addressed the structural and pharmacological limitations of Thalidomide by synthesizing analogs using benzothiazole molecules.

Recently, benzothiazoles have gained considerable interest thanks to their anti-tumor, anti-inflammation and anti-microbial properties (Ali and Siddiqui, 2013; Keri et al., 2015). A category of benzothiazoles called 2-amino-benzothiazoles is often fused to phthalic anhydride to obtain phthalimide derivatives that do not contain chiral carbons and do not have multiple enantiomers (Kok et al., 2008). Therefore, in this study four phthalimide derivatives, analogs of Thalidomide have been synthesized and tested for their anti-angiogenic functions using a 3D angiogenesis cell culture assay. Following this, the most effective compound in the *in vitro* studies was selected for an *in vivo* screening of its possible teratogenic effects in a zebrafish embryo model.

Although a large number of new treatments are developed and reach the clinic, there is no improvement on survival rate, probably due to the lack of appropriate models used for pre-clinical testing (Rodenhizer et al., 2018). Keeping in mind the ongoing challenges faced during clinical trials, both our *in vitro* and *in vivo* assays have been designed for optimal pre-clinical testing of the compounds.

*In vitro* 3D microfluidic platforms offer a series of advantages compared to the standard *in vitro* system for drug screening. They enable a reduction in reagents and costs and the possibility to generate cytokine and chemokine gradients. Most importantly, they recapitulate the three-dimensional tissue architecture and cell-cell or cell-matrix interactions during biological processes (Adriani et al., 2016a, b, c; Lee et al., 2018) Furthermore, microfluidic devices allow researchers to impose different oxygen concentration levels and the possibility to co-culture multiple cell types in 3D (Adriani et al., 2015; Pavesi et al., 2015; Pavesi et al., 2017) to better mimic physiological conditions found *in vivo* compared to standard 2D culture systems. Microfluidic devices are compatible with various microscopy techniques for real-time imaging of the biological processes due to the transparency of materials they are made up of and offer the possibility to retrieve cells and supernatants to perform downstream biochemical assays to analyze differences in gene and protein expression.

Similarly, zebrafish larvae are an ideal model for screening both angiogenic and teratogenic functions. They have been successfully used for anti-angiogenic drug screening for over 15 years (Serbedzija et al., 1999; Beedie et al., 2016; Chávez et al., 2016). Their vasculature develops rapidly and can be easily monitored due to the optical transparency of the zebrafish embryos. In our study, we used the *fli1a*:EGFP transgenic zebrafish line that drives expression of the enhanced green fluorescent protein (EGFP) in endothelial cells for a better visualization and assessment of the vasculature (Lawson and Weinstein, 2002).

Moreover, Thalidomide is not teratogenic in other common vertebrate animal models such as mice and rats (Miller and Strömland, 1999; Melchert and List, 2007; Knobloch and Rüther, 2008) while it has been shown to mediate anti-angiogenic and teratogenic actions in zebrafish (Therapontos et al., 2009; Ito et al., 2010).

## Materials and Methods

### Synthesis of Phthalimide Derivatives

Thalidomide was purchased from Sigma-Aldrich (CAS number: 50351). 2-aminobenzothiazole (CAS number: 108812) and 2-amino-6-methylbenzothiazole (CAS number: 288373) were purchased from Sigma-Aldrich. Phthalic anhydride and 4-methyl- phthalic anhydride were purchased from Lancaster. Commercial 2-aminobenzothiazoles (2a, b) were fused with phthalic (for C1–C2) or 4- methylphthalic (for C3-C4) anhydride as reported in the literature (Pawar et al., 2014). The structure of target compounds was assigned by GC/MS, ^1^H NMR, ^13^C NMR, IR, elemental analysis.

The synthesis of substituted 2-(benzo[d]thiazol-2-yl)isoindoline-1,3-dione (C1) was adapted from Pawar et al. (2014) and briefly consisted of heating equimolar quantities of phthalic anhydride and 2-aminobenzothiazole (2a in Scheme 1 in Supplementary Material). The mixture was occasionally stirred, and phthalic anhydride which sublimed was pushed down into the reaction mixture, until the fusion was completed. The mixture was kept undisturbed for 5 min to allow the liquid mass to solidify. The solid mass was suspended in water to remove unreacted anhydride. The residue was washed with a mixture of water and 96% ethanol (in a ratio 1:1) at a high temperature and filtered. The white solid obtained was recrystallized with methanol/chloroform/acetone to give white crystals. 1b was prepared as reported for 1a by reacting 6-methyl-2 aminobenzothiazole (2b in Scheme 1 in Supplementary Material) with phthalic anhydride. 1c was prepared as reported for 1a by reacting 2-aminobenzothiazole (2a in Scheme 1 in Supplementary Material) with 4-methylphthalic anhydride. 1d was prepared as reported for 1a by reacting 6-methyl -2 aminobenzothiazole (2b in Scheme 1 in Supplementary Material) with 4-methylphthalic anhydride.

For simplicity, the compounds are referred to as C1 (1a), C2 (1b), C3 (1c) and C4 (1d) and the complete characterization of the compounds can be found in the Supplementary Material.

### Preparation of Thalidomide and Compound Solutions

Thalidomide and its as-synthesized analogs were dissolved in dimethylsulfoxide (DMSO) at room temperature to make stock solutions. We used 0.0001% DMSO for C4 at 0.02 μM, 0.0005% DMSO for C4 at 0.1 μM, 0.002÷0.0024% DMSO for all compounds at 0.5 μM, 0.11÷0.013% DMSO for all compounds at 25 μM, 0.21% for Thalidomide at 50 μM and 0.43÷0.5% DMSO for compounds at 100 or 200 μM. Before each experiment, stock solutions were filtered with centrifuge tube filter and the final concentration was obtained by dilution of the stock solution.

#### Cell Culture

Human Umbilical Vein Endothelial Cells (HUVECs, C2517AS, Lonza, Basel, Switzerland) were cultured and expanded in standard T75 culture flasks containing in endothelial growth medium-2 (EGM-2, Lonza) and kept in incubators at 37°C and 5% CO_2_. HUVECs, at passages between 4 and 6 with about 80% confluence were used in all the experiments.

#### *In vitro* Cytotoxicity Assay

Thalidomide and the compounds were tested by means of a CellTox^TM^ Green Cytotoxicity Assay (Promega Corporation, Cat. N. G8741) to evaluate their cellular cytotoxicity. For the assay, HUVECs were seeded in black 96-well plates at 1 x 10^3^ cells/well in EGM-2 and allowed to attach overnight at 37°C and 5% CO_2_. The culture medium was then aspirated and fresh culture medium containing one of the four compounds or Thalidomide was added to each well. HUVECs were treated with Thalidomide and compounds at three different concentrations, namely 100, 25, and 0,5 μM. After 72 h, the CellTox Green reagents were added, the plate was shaken and incubated at room temperature for 15 min covered from light. The fluorescence signal produced by the dye specifically binding to the dead-cell DNA was measured using an Infinite M200 Pro Tecan (Life Sciences) plate reader and considered proportional to compound cytotoxicity.

### Cell Viability Assay in the Microfluidic Device

Viability of HUVECs in the device, after 72 h of treatment with each compound at 100 μM, was assessed by staining the cell nuclei with Hoechst 33342 (NucBlue^®^ Live ReadyProbes, Cat.No R37605, Life Technologies) and Nuc Green dye that is specifically able to bind DNA of dead cells (NucGreen^®^ Dead 488 ReadyProbes^®^ Reagent, Cat. No R37109, Life Technologies). HUVECs treated for 72 h with 0.5% DMSO were used as control. HUVECs were fixed with 4% paraformaldehyde for 15 min at room temperature and z-stack images for each condition were acquired with a confocal microscope (LSM 710; Carl Zeiss, Thornwood, NY, United States). Imaris software (Bitplane, Zurich, Switzerland) was used to analyze the images and quantify the number of live and dead cells with the function spots. The percentage of live cells was calculated with the following formula:

$$\%livecells=\frac{\left(\#\,t\,o\,t\,a\,l\,c\,e\,l\,l\,s-\#\,d\,e\,a\,d\,c\,e\,l\,l\,s\right)}{\#\,t\,o\,t\,a\,l\,c\,e\,l\,l\,s}*100$$

### *In vitro* 3D Microfluidic Angiogenic Assay

Microfluidic plastic chips and holders were purchased from AIM Biotech company (AIM Biotech, Singapore). Each chip contains three devices and each device has three channels: a central channel able to host a hydrogel and two lateral fluidic channels for culture media and endothelial cells. The hydrogel was prepared from collagen type I solution (Corning) at 2 mg/ml following the Aim Biotech Protocol. The hydrogel was gently pipetted into the middle channel of the devices and polymerized for 30 min at 37°C and 5% CO_2_. Fibronectin coating of the microchannels was performed to improve cell adhesion. Fibronectin (Sigma- Aldrich, Cat. No. F0895) was diluted in 1x Phosphate buffer saline (PBS - Life Technologies) at a final concentration of 50 μg/ml, injected into the device and incubated for 1 h at 37°C and 5% CO_2_ (Shin et al., 2012). HUVECs were subsequently seeded in one of the lateral fluidic channels at 3 × 10^6^ cell/ml. After 24 h, VEGF (Recombinant Human Protein from Thermo Fisher- PHC9394) at a final concentration of 40 ng/ml and Sphingosine-1-Phosphate, S1P (Sigma-Aldrich, Cat.No. S9666) at a final concentration of 125 nM together with one of the compounds were added in the channel containing endothelial cells (Lim et al., 2013). Together with the VEGF and S1P treatment, an interstitial flow due to a hydrostatic pressure gradient was applied to promote HUVECs angiogenic sprouting in the hydrogel within 3 days. Each compound C1–C4 and Thalidomide were tested in the microfluidic angiogenic assay at three different concentrations, 100, 25, and 0.5 μM. The treatment was maintained for 72 h. The devices were kept in incubators at 37°C and 5% CO_2_, the medium was changed every 24 h and cell sprouting was monitored daily by a phase-contrast microscope (Olympus CKX41, Europe).

### Quantification of Cell Sprouting

After 72 h of culture in the device, the endothelial cells were fixed with 4% paraformaldehyde for 15 min at room temperature and permeabilized with 0.1% Triton X-100 for 12 min. The cells were incubated for 25 min at room temperature with an F-actin probe conjugated to the fluorescent dye tetramethylrhodamine (TRITC) (ActinRed^TM^ 555 ReadyProbes^®^, Cat. No. R37112, Life Technologies) and with Hoechst 33342 (NucBlue^®^ Live ReadyProbes, Cat.No R37605, Life Technologies).

Confocal microscopy was used to capture z-stack images of the angiogenic sprouting. Imaris software (Bitplane) was used to analyze the acquired images to quantify the volume of the 3D angiogenesis network, the length of the sprouting filaments and the branch levels. For clarity, by software definition, the branch level at the filament origin is 0 and a branching point is considered a point where a single filament spreads in two other segments. After each branching point, the branch level increase of 1.

### *In vivo* Zebrafish Model

*Danio rerio* (zebrafish) maintenance and breeding was carried under standard conditions with a 14-h light/ 10-h dark cycle at 28.5°C. Adult wild-types (*AB*) and *fli1a*:EGFP transgenic zebrafish were pair-mated in breeding tanks. Embryos were sorted from unfertilized eggs and left to develop in E3 medium at 28°C. All experiments with zebrafish embryos were approved by the Singapore National Advisory on Laboratory Animal Research.

### Drug Treatment *in vivo*

Zebrafish embryos were dechorionated before drug application at 12 h post fertilization (hpf). For Thalidomide treatment, a 800 μM stock warmed solution was added to a final concentration of 50, 100, and 200 μM. For C4 treatment, a 100 μM stock solution was added to a final concentration of 0.02, 0.1, 0.5, and 5 μM and the solutions were added to E3 medium as described previously. The concentrations tested for C4 were much lower than the concentrations tested for Thalidomide because we observed in the *in vitro* experiments that C4 was very effective already at a concentration of 0.5 μM; thus, we decided to further reduce its concentration in the *in vivo* treatment. The medium was replaced every 12 h with freshly prepared drug solutions. Embryos were anesthetized using Tricaine mesylate (50 mg/ml) and fixed overnight in Fish Fix (0.1 M sodium phosphate buffer, 12 mM CaCl2, 4% paraformaldehyde, 4% sucrose) at 30 and 75 hpf. Tissue was equilibrated via a series of washes in 25, 50, and 75% glycerol/water. Images were acquired by confocal microscopy (LSM 710; Carl Zeiss, Thornwood, NY, United States).

### Measurement of Stalks Length

Seventy-five post fertilization embryos were fixed and mounted in lateral view and imaged with Zeiss confocal. To quantify the anti-angiogenic effect of the drug treatment, the length and the number of intersegmental vessels of the zebrafish tail were quantified using Imaris Software. The intersegmental vessels of the zebrafish tail were also divided in three groups (proximal: 1–5; intermediate: 6–10 and distal 11–15) to observe any difference in the effect of treatment along the zebrafish tail.

### Measurement of Teratogenic Parameters: Pectoral Fin, Otic Vesicle Size and Eye Area

For the pectoral fin and otic vesicle a fluorescent stereo microscope (Leica M205 FA) was used to acquire images of the embryos in either lateral or dorsal view. The length of the pectoral fin was measured at 75 hpf while the length of the long axis of the otic vesicle was measured at 30 hpf using Imaris Software. The area of the zebrafish eye was measured from the confocal images and calculated following the formula considering the eye as an ellipsoid:

$$A=\max d\,i\,a\,m^{*}\,\min d\,i\,a\,m^{*}\,\pi$$

### Statistics

At least three devices per condition and three regions of interest (ROI) per device were considered. Similar for the *in vivo* assays, at least three embryos per condition were considered. Analysis was conducted using Prism 6.0 (GraphPad Software, La Jolla, CA) and statistical significance was assessed using ANOVA with multiple comparisons.

## Results

### Thalidomide and Phthalimide Derivatives Treatment Are Not Cytotoxic to Endothelial Cells

**FIGURE 1:**
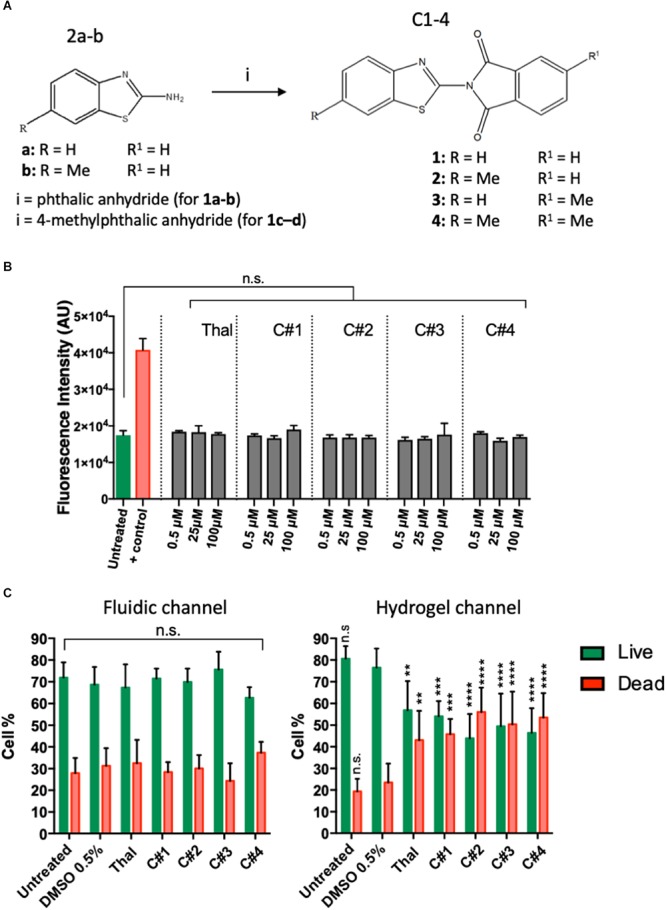
Chemical structures of the new phthalimide derivatives and *in vitro* cytotoxicity tests. **(A)** 2-aminobenzothiazoles (2a) or 6-methyl -2 aminobenzothiazole (2b) reacted with phthalic anhydride (for compound C1–C2) or with 4-methylphthalic anhydride (for compound C3-C4) to obtain the final compounds constituted by a benzothiazolic portion and a phthalic portion. **(B)** Cytotoxicity assay was performed on human endothelial cells treated or untreated for 72 h with one compound or Thalidomide at 100, 25, and 0,5 μM. 100% DMSO was used as positive control. The plots show means ± SD of the fluorescent intensity for three wells for each condition. Statistics were calculated by one-way ANOVA. **(C)** Viability of human endothelial cells in the device was tested after 72 h treatment with each compound at 100 μM. Treatment with vehicle was used as control. The data represent the mean ± SD of the % of live (green) or dead (red) cells for three regions of interest per condition. Statistics were calculated by two-way ANOVA. n.s. = not significant; **p < 0.01; ***p < 0.001; ****p < 0.0001.

Human endothelial cells were treated for 72 h with the four synthesized compounds (C1–C4, Figure 1A) or Thalidomide to determine the possibility of cytotoxic effects. Each compound was tested at three different concentrations, 100, 25, and 0.5 μM. We monitored changes in membrane integrity using the CellTox^TM^ Green Cytotoxicity Assay. During cell death, the breakdown of cell and nuclear membranes causes nuclear DNA to become accessible for dye-binding. Fluorescence was quantified and considered proportional to cytotoxicity. At all analyzed concentrations, Thalidomide as well as all compounds C1–C4 maintained a fluorescent intensity similar to the untreated cells. A positive control (100% DMSO) was used to confirm that the assay was able to identify dead cells (Figure 1B). This suggests that the drug treatment had no cytotoxic effects on endothelial cells.

### Treatment With Thalidomide and Phthalimide Derivatives Decreases Endothelial Cells Viability in the Hydrogel Channel

Human endothelial cells cultured in the microfluidic device were labeled for live and dead cells to assess their viability after of treatment with Thalidomide, compounds C1–C4 or with vehicle. Confocal images were acquired and analyzed in IMARIS. The spot tracking function was used to quantify the number of live and dead cells in the microfluidic platform to calculate the percentage of live and dead cells into the channel of the device after treatment. Figure 1C shows the percentage of live and dead cells into the medium or hydrogel channels after treatment with Thalidomide and compounds C1–C4, at the concentration of 100 μM. Statistical analysis did not show significant differences between the percentage of live or dead cells in the fluidic channel containing the endothelial cells and medium for the untreated, vehicle control or compound conditions. Interestingly, significant differences were observed in the hydrogel channel where cells treated with either Thalidomide or compounds C1–C4 were less viable than untreated or vehicle control cells. These results could suggest that the compound presents cytotoxic effects only in the cells migrating and sprouting into the hydrogel.

### Phthalimide Derivatives Are More Effective Than Thalidomide in Reducing Angiogenesis *in vitro*

**FIGURE 2:**
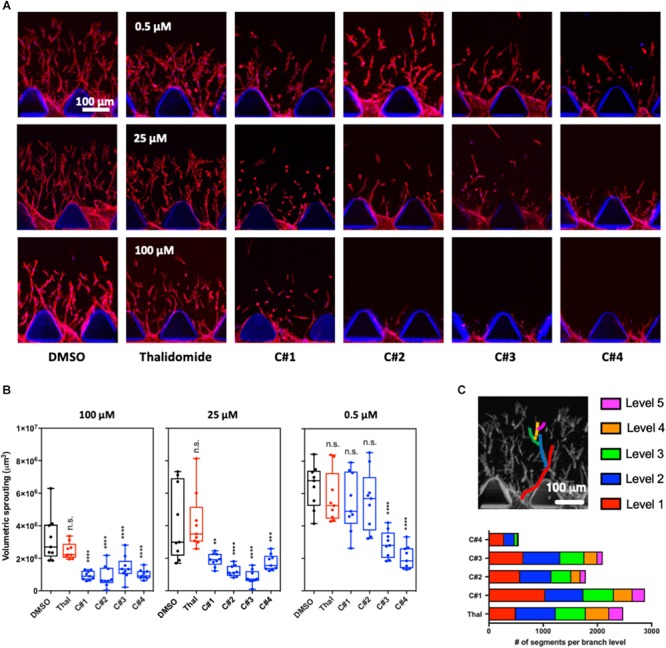
3D angiogenesis assay in microfluidic device. **(A)** Representative confocal images of the angiogenic sprouting in the 3D extracellular matrix-like hydrogel. Human endothelial cells were treated with Thalidomide or one of the compounds at 0.5, 25, and 100 μM for 72 h. Treatment with vehicle was used as control. Cells are stained for F-actin and Hoechst to visualize actin cytoskeleton filaments and nuclei, respectively. **(B)** Analysis of the volumetric sprouting for each condition. The box and whiskers plots show all the data points quantified for at least three devices per condition. Statistics were calculated by one-way ANOVA. **(C)** The different network complexity for each compound or for Thalidomide was analyzed by quantifying the branch levels and the number of segments for each branch level combining all the concentrations for each compound. n.s. = not significant; **p < 0.01; ***p < 0.001; ****p < 0.0001.

We measured endothelial cell sprouting in the device after 72 h of treatment for each condition (Figure 2A). Three main parameters were considered: sprouting volume, number of branch levels and associated number of segments per branch level. Volumetric sprouting was significantly reduced for all the compounds C1–C4 at higher concentrations (25 and 100 μM) (Figure 2B). However, only the compounds C3 and C4 showed a significant reduction of the sprouting volume at the lowest concentration (0.5 μM).

The branch level analysis was used to assess the complexity of the angiogenic network and all the tested concentration for each drug were combined in the final plot (Figure 2C). During angiogenesis, cells generate filaments that can separate in two or more segments contributing to the formation of a complex network of capillaries. The more branching points and levels are present, the more complex the angiogenic network is. In our experiments, up to five branch levels were observed Thalidomide and compounds C1, C2, and C3 treatment greatly reduced the number of segments from level 1 to level 5 (Figure 2C). Strikingly in cells treated with C4, not only was the number of segments at each level substantially reduced, but there was a complete loss level 5 segments (Figure 2C). These results suggest that compound C4 has the highest potency in preventing the formation of a complex angiogenic networks among the tested compounds.

### C4 Phthalimide Derivative Retains Its Anti-angiogenic Activity *in vivo*

**FIGURE 3:**
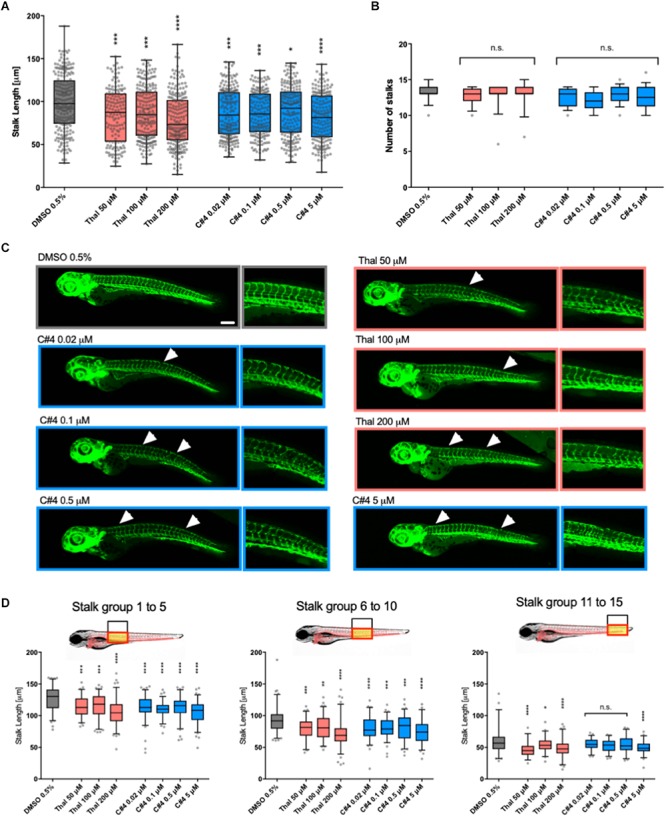
Quantification of the anti-angiogenic effects in zebrafish embryos. Stalks length **(A)** and number **(B)** were measured for *fli1a*:EGFP zebrafish embryos at 75hpf treated with vehicle, Thalidomide or compounds at different concentrations. The box and whiskers plots show the data points quantified for at least ten embryos per condition. **(C)** Representative confocal images of zebrafish for each condition. White arrows show a missing stalk, a reduction in vessel length or loss of vascular connectivity. **(D)** The length of stalks was plotted here for three different groups of stalks. Statistics were calculated by one-way ANOVA. n.s. = not significant; *p < 0.05; ^∗∗^*p* < 0.01; ***p < 0.001; ****p < 0.0001.

To confirm the anti-angiogenic capability of our novel Thalidomide analog C4, we tested its functions *in vivo*. Specifically, we used *fli1a*:EGFP zebrafish transgenic line, which express green fluorescent protein (GFP) on endothelial cells during development, as a tool for visualizing developing blood vessels (Lawson and Weinstein, 2002). Developing embryos were treated at 12 hpf with either Thalidomide, compound C4 or a control (DMSO 0.5%). Following previous literature, Thalidomide was used for the treatment at concentrations of 50, 100, and 200 μM, while C4 treatment was tested at 5, 0.5, 0.1, and 0.02 μM concentrations. The embryos were fixed at 75 hpf to analyze the effects of these compounds on vessel development. With both Thalidomide and C4, we observed a reduction of the stalk length confirming their anti-angiogenic activity at all tested concentrations (Figure 3A). Importantly, C4 concentrations were much lower than Thalidomide concentrations suggesting an increased potency as an anti-angiogenic drug. After treatment with compound C4 we often observed a reduction in the total number of stalks per embryo although there was no statistically significant difference compared with the vehicle (Figure 3B). Representatives images of GFP vasculature in our zebrafish model (Figure 3C) highlight the effect of C4 as anti-angiogenic compound at lower doses compared to Thalidomide. White arrows in Figure 3C indicates remarkable vasculature modifications. Figure 3D shows that, considering three groups along the zebrafish tail from proximal to distal, the length of the intersegmental vessels is more affected by C4 treatment in the proximal and intermediate portions than in the distal one.

### Effective Anti-angiogenic Concentrations of C4 Present a Reduced Teratogenic Effect Compared to Thalidomide *in vivo*

**FIGURE 4:**
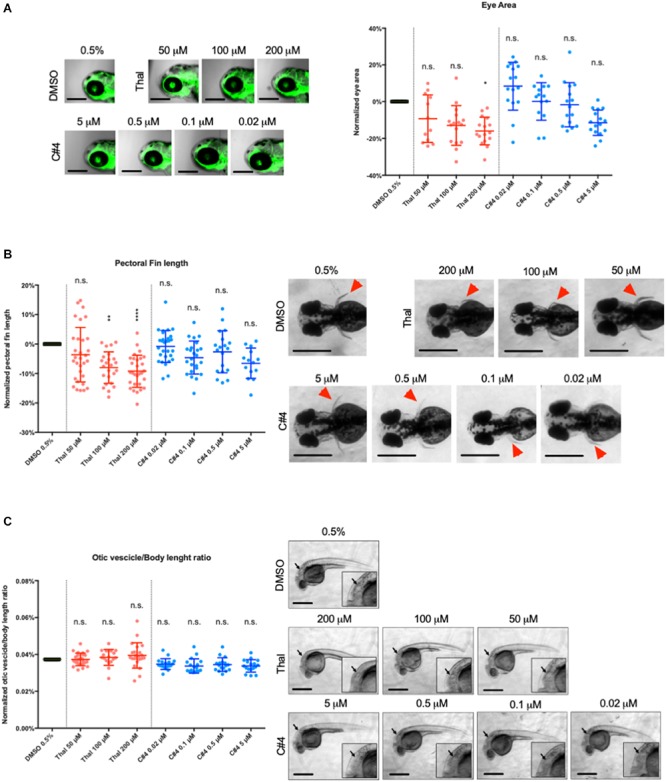
Quantification of the teratogenic side effects in zebrafish embryos. **(A)** Malformations were measured by three teratogenic parameters in the zebrafish embryo: the eye area **(A)**, the pectoral fin length **(B)** and the ratio between the long axis of the otic vesicle and the body length **(C)**. The eye area **(A)** and the pectoral fin **(B)** were measured at 75hpf and the otic vesicle **(C)** was measured at 30hpf for optimal visualization. Lateral view of embryo eye **(A)**, dorsal views of pectoral fins (red arrowheads in **B**) and lateral view of the otic vesicle for each condition are showed. For each parameter, the data are shown as percentage variation in respect to the treatment with vehicle. The data are plot in a scattered dot plot showing the mean ± SD. Statistics were calculated by one-way ANOVA. n.s. = not significant; *p < 0.05; **p < 0.01; ****p < 0.0001.

Zebrafish larvae were screened for potential teratogenic effect from Thalidomide and C4 drug treatment. We first measured the size of the otic vesicle of zebrafish embryos at 30 hpf. Then, eye area and pectoral fin length were subsequently measured in zebrafish embryos at 75 hpf. All the parameters are expressed as percentage variation with respect to the vehicle control. The mean eye area decreased after treatment with Thalidomide although the difference was only statistically significant for 200 μM (Figure 4A). The mean eye area remained relatively unaffected after C4 treatment with a slight reduction at 5 μM (Figure 4A). The pectoral fin length was drastically reduced after treatment with Thalidomide at both 100 and 200 μM while there were no changes in the zebrafish larvae treated with varying concentrations of the C4 compound (Figure 4B). Finally, the otic vesicle sizes were not affected by treatment with either Thalidomide or C4 at all tested concentrations (Figure 4C).

## Discussion

The limitations of current anti-cancer chemotherapy are motivating Scientists to design and synthesize new molecules with the ability to inhibit tumor progression whilst keeping minimal side effects. For this purpose, angiogenesis is an ideal target because vascularized tumors receive the blood supply necessary to grow and metastasize (Benouchan and Colombo, 2005). In this study we present four Thalidomide analogs (Figure 1A) with the aim to identify a safer and more effective anti-angiogenic compound. Thalidomide was chosen as a reference drug for its known anti-angiogenic properties (Kenyon et al., 1997; Komorowski et al., 2006) as well as its severe teratogenic effects (Miller and Strömland, 1999; Melchert and List, 2007; Knobloch and Rüther, 2008; Therapontos et al., 2009). Although, other Thalidomide analogs have been synthesized, such as Lenalidomide and Pomalidomide, they were found to have neurotoxic effects (Patel et al., 2015) and also present a chemical structure that, like Thalidomide, includes a chiral carbon. For this reason, Lenalidomide and Pomalidomide were not considered in the present study even if future steps in the validation of our compounds could include a direct comparison with them. The compounds were designed to have a benzothiazole portion (Shi et al., 1996; Bradshaw et al., 1998) and a phthalimide portion eliminating the presence of the chiral carbon, known to be a plausible cause for teratogenicity (Miller and Strömland, 1999; Chaudhry et al., 2002; Hanje et al., 2006; Melchert and List, 2007; Ito et al., 2010; Mercurio et al., 2017). Phthalimide, an imido derivative of phthalic acid, consists of two carbonyl groups bound to nitrogen. This compound is structurally related to acid anhydrides and its hydrophobicity and neutrality allow membrane-permeability *in vivo* (Al-Azzawi and Al-Razzak, 2014). Phthalimide, together with N-substituted phthalimides, represents an important class of compounds that possess important biological activities including anti-inflammatory (Machado et al., 2005), analgesic (Antunes et al., 1998) and hypolipidemic (Sena et al., 2003) functions. Further, the fusion between phthalimide and 2-amino-benzothiazole, has already led to the discovery of new compounds with cytotoxic anti-tumor properties. For example, Kini et al. (2007) synthesized 2- aryl substituted benzothiazoles derivatives and evaluated them against human cervical cancer cell lines. In addition, Kok et al. (2008) demonstrated that presence of a benzothiazole unit attached to the nitrogen of phthalimide exhibits *in vitro* cytotoxic activity on human cancer cell lines with both caspase-dependent and -independent pathways involved in cancer cells apoptosis. Another study by Nagarajan et al. (2013) discovered the anti-angiogenic property of benzimidazole containing phthalimide derivatives using yolk angiogenesis model. These studies support the potential biological and pharmaceutical activity of our four compounds obtained by summing the characteristics of phthalimide and 2-amino benzothiazole with two different substituents, H and CH${}_{3}$, on each portion.

Firstly, we verified that compounds C1, C2, C3, C4 and Thalidomide were not cytotoxic for human endothelial cells. Then, we tested their potential anti-angiogenic properties in a 3D microfluidic angiogenesis assay. The assay consisted of culturing endothelial cells in a fluidic channel adjacent to an extracellular matrix-like hydrogel to mimic the endothelial cell sprouting in 3D. An interstitial flow and a chemical stimulation with VEGF and S1P were applied in order to promote the cell sprouting into the collagen hydrogel. The microfluidic platform allowed us to efficiently screen the cell response to each treatment. After 3 days of treatment, the cells were stained and imaged to analyze the angiogenic sprouting as well as cell viability. In the fluidic channel containing the endothelial cells and medium, the treated cells presented a percentage of live and dead cells similar to the untreated cells or to the cells treated with the highest concentration of DMSO used to dissolve the compounds (0.5%), confirming the lack of cytotoxic activity for all the compounds and Thalidomide (Figure 1B). Interestingly, cells treated in the hydrogel channel showed a higher percentage of dead cells compared to the untreated cells suggesting that the treatment with either compounds or Thalidomide may have affected only sprouting cells. Further investigations on the mechanisms of action are needed to verify this hypothesis.

The analysis of the sprouting in our 3D angiogenesis assay showed that angiogenesis is significantly inhibited after treatment with all compounds at concentrations higher than 25 μM while Thalidomide treatment at all three concentration seemed to have no significant effect. Further, C3 and C4 presented an anti-angiogenic function even at the lowest concentrations tested (Figures 2A,B). Additionally, C4 treatment dramatically reduced the number of segments for each branch level representing a decrease in network complexity (Figure 2C). These results clearly demonstrate that all the tested compounds have an anti-angiogenic function at a much lower effective dose compared to Thalidomide. These results also suggested that the presence of substituents in the chemical structures of each compound plays a role in their inhibitory activity: although, all compounds produced a reduction in angiogenesis in terms of cell sprouting, compound C4, containing a methyl group on both the phthalic and the benzothiazolic portions of the molecule, was found to be the most effective. The methyl group has a considerably greater steric bulk compared to hydrogen group and we could speculate that this may have played a role in the increased anti-angiogenic activity of C4 compared to the standard drug or to the other compounds. As a drawback, the presence of a methyl group decreases the polarity of the molecule and accounts for the limited solubility in water of all these compounds. This is particularly true for compound C4 which contains two methyl groups.

The quantitative data from our 3D *in vitro* angiogenic assay allowed us to identify C4 as the most effective compound. Thus, C4 was subsequently used for *in vivo* analysis. Its anti-angiogenic potency and teratogenic side effects were tested in a vertebrate animal model. The teratogenic activity of Thalidomide exhibits species specificity and commonly available rodent models were shown to be inappropriate to study Thalidomide teratogenic side-effects (Nau, 1986; Franks et al., 2004; Vargesson, 2013). Therefore, zebrafish (Danio rerio) were chosen as they have previously been used to study teratogenic side effects. Further, zebrafish embryos are easier to handle than other vertebrates such as rabbits or chicks, as they develop rapidly and their optical transparency facilitates the observation of internal blood vessel development.

*In vivo* experiments were conducted with Thalidomide as a positive control at concentrations of 50, 100, and 200 μM following previous studies showing teratogenic effects for concentrations higher than 100 μM (Yabu et al., 2005; Ito et al., 2010; Beedie et al., 2016). The treated samples were compared with 0.5% DMSO that is the highest DMSO concentration used to dissolve the compounds to have the most conservative results. Otic vesicle size, pectoral fin length and eye area were used as parameters for the evaluation of the drug teratogenic activity while the stalk number and the blood vessel length were used to quantify the drug anti-angiogenic function. As a result, we showed that compound C4 is very effective as an anti-angiogenic agent for all the concentrations tested ranging from 0.02 to 5 μM. Thalidomide had similar anti-angiogenic properties to C4 but at much higher concentrations (from 50 to 200 μM). After treatment with Thalidomide at 200 μM, there was a reduction in eye size. At both 100 or 200 μM, we observed a significant reduction of the pectoral fin length in agreement with the data by Ito et al. (Ito et al., 2010) This finding is reminiscent of the birth defects observed in children of mothers treated with Thalidomide and reinforces the conservation of developmental pathways between teleosts and humans. In addition, after Thalidomide treatment, we often observed cardiac edema, confirming its teratogenic activity. The otic vesicle, the pectoral fin length and the eye area were not affected by C4 treatment at any of the tested concentrations.

In conclusion, our *in vitro* results suggest that all the synthetic Thalidomide analogs present a much lower effective dose with an anti-angiogenic function compared to Thalidomide and that C4 is the most effective. *In vivo*, C4 showed an enhanced anti-angiogenesis potency with reduced side effects compared to Thalidomide. Further investigation to elucidate the molecular pathways activated by C4 would be important for downstream drug development. This could involve investigating if Cereblon, the molecular target of Thalidomide and its chiral analogs Lenalidomide and Pomalidomide (Ito et al., 2010; Lopez-Girona et al., 2012), is also the molecular target of C4.

## Ethics Statement

This study was carried out in accordance with the recommendations of the Singapore National Advisory on Laboratory Animal Research. The protocol was approved by the Singapore National Advisory on Laboratory Animal Research.

## Author Contributions

GA, AP, RK, AM, and LS conceptualized the experimental assays. GA, AM, and LS performed the methodology. AM acquired the data. AM, GA, and AP analyzed the data. AM, ACat, and ACar performed the chemical synthesis. AM, GA, AP, and LS wrote the manuscript. CF, FC, and RK provided the resources. GA, AP, RK, FC, and AV supervised the study.

## Conflict of Interest Statement

Experiments were conducted in a microfluidic device marketed by AIM Biotech Pte. Ltd, a company in which RK has a significant financial interest as cofounder and board member. AP is a consultant for AIM Biotech Pte. Ltd. The remaining authors declare that the research was conducted in the absence of any commercial or financial relationships that could be construed as a potential conflict of interest.
