# Supplementary material for: Phthalimide Derivative Shows Anti-angiogenic Activity in a 3D Microfluidic Model and No Teratogenicity in Zebrafish Embryos
Source: Front Pharmacol. 2019 Apr 17;10:349. doi: 10.3389/fphar.2019.00349 (PMC6479179; doi:10.3389/fphar.2019.00349)
Supplement: Supplementary file 1 [file Table_1.docx]

Supplementary Material

**Compound chemical characterization**

Compounds **C1-4** were synthesized as depicted in Figure 1A. The structures of the compounds were confirmed by standard spectrometric and spectroscopic analyses. Melting points were determined by a Gallenkamp apparatus in open glass capillary tubes. Infrared spectra were recorded by a Perkin-Elmer (Norwalk, CT) Spectrum One FT spectrophotometer and band positions are given in cm^–1^. ^1^H NMR and ^13^C NMR spectra were recorded on an Agilent 500 MHz operating at 500 and 125 MHz for ^1^H and ^13^C, respectively, or on a Varian VX Mercury spectrometer operating at 300 and 75 MHz, for ^1^H and ^13^C, respectively, using CDCl_3_ and DMSO-*d_6_* as solvents. Chemical shifts are reported in parts per million (ppm) relative to the residual non-deuterated solvent resonance: CDCl_3_, 7.26 (^1^H NMR) and 77.3 (^13^C NMR); DMSO-*d*_6_, 2.48 (^1^H NMR) and 40.3 (^13^C NMR). *J* values, which quantify the number and the size of couplings, are given in Hz. Gas chromatography (GC)/mass spectroscopy (MS) was performed on a Hewlett-Packard 6890–5973 MSD at low resolution. Elemental analyses were performed on a Eurovector Euro EA 3000 analyzer. Chromatographic separations were performed with silica gel columns by column chromatography (Kieselgel 60, 0.040–0.063 mm, Merck, Darmstadt, Germany). Thin layer chromatography (TLC) analyses were performed with pre-coated silica gel on aluminum sheets (Kieselgel 60 F_254_, Merck).

2-(Benzo[d]thiazol-2-yl)isoindoline-1,3-dione (**C1**)

Yield: 26%; white crystals; mp 249–250 °C (methanol/CHCl_3_/acetone); **GC/MS** (70 eV) m/z (%): 312 (M+, 100); **IR** (KBr): 1724 (C=O) cm–1; **^1^H NMR** (300 MHz, CDCl_3_): *δ* 7.44 (t, *J =* 7.6 Hz, 1H, Ar), 7.54 (t, *J =* 7.3 Hz, 1H, Ar), 7.87–7.93 (m, 3H, Ar), 8.05–8.08 (m, 2H, Ar), 8.15 (d, *J =* 8.3 Hz, 1H, Ar); **^13^C NMR** (75 MHz, CDCl_3_): *δ* 121.2 (1C), 123.4 (1C), 124.6 (2C), 125.4 (1C), 126.4 (1C), 131.1 (2C), 132.9 (2C), 135.4 (1C), 149.5 (1C), 151.9 (1C), 164.5 (2C). Anal. calcd for C_15_H_8_N_2_O_2_S (280.03): C 64.27; H 2.88; N 9.99. Found: C 63.98; H 2.87; N 9.95.

2-(6-Methyl-1,3-benzothiazol-2-yl)-1H-isoindol-1,3(2H)-dione (**C2**)

Yield: 74%; Yellow crystals; mp>250 ˚C (CHCl_3_/hexane); **GC/MS** (70eV) m/z (%): 294 (M+, 100): **IR** (KBr): 1793, 1729 (C=O) cm^–1^; **^1^H NMR** (300 MHz, CDCl_3_): δ2.50 (s, 3H, C*H*_3_), 7.32 (dd, *J =* 8.3, 1.5 Hz, 1H, Ar), 7.68 (s, 1H, Ar), 7.83–7.88 (m, 2H, Ar), 8.0 (d partially overlapping on m 8.0–8.06 ppm, *J =* 8.3 Hz, 1H, Ar), 8.0–8.06 (m partially overlapping d at 8.0 ppm, 2H, Ar); **^13^C NMR** (75 MHz, CDCl_3_): δ 21.6 (1C), 120.8 (1C), 122.9 (1C), 124.5 (2C), 128.0 (1C), 131.1 (1C), 133.1 (1C), 135.3 (2C), 135.6 (2C), 147.5 (1C), 150.9 (1C), 164.6 (2C). Anal. calcd for**:** C_16_H_10_N_2_O_2_S (294.33): C 65.29; H 3.42; N 9.52. Found: C 65.13; H 3.56; N 9.61.

2-(1,3-Benzothiazol-2-yl)-5-methyl-1H-isoindole-1,3(2H)-dione (**C3**)

Yield: 78%; Yellow crystals: mp 210–212 °C (CHCl_3_/hexane); **GC/MS** (70 eV) m/z (%): 294 (M^+^, 100); **IR** (KBr): 1789, 1727 (C=O) cm^–1^; **^1^H NMR** (500 MHz, CDCl_3_): *δ* 2.56 (s, 3H, C*H*_3_), 7.40 (t, *J* = 15.2 Hz, 1H, Ar), 7.50 (t, *J* = 14.7 Hz, 1H, Ar), 7.63 (d, *J* = 7.3 Hz, 1H, Ar), 7.81 (s, 1H, Ar), 7.89 (t, *J* = 14.7 Hz, 2H, Ar), 8.11 (d, *J* = 7.8 Hz, 1H, Ar); **^13^C NMR** (125 MHz, CDCl_3_): *δ* 22.2 (1C), 121.1 (1C), 123.3 (1C), 124.5 (1C), 125.0 (1C), 125.3 (1C), 126.4 (1C), 128.4 (1C), 131.4 (1C), 132.8 (1C), 136.0 (1C), 147.1 (1C), 149.5 (1C), 152.0 (1C), 164.5 (1C), 164.6 (1C). Anal. calcd for C_16_H_10_N_2_O_2_S 0.33H_2_O (300.05): C 63.99; H 3.58; N 9.33. Found: C 64.26; H 3.43; N 9.40.

2-(6-Methyl-1,3-benzothiazol-2-yl)-5-methyl-1H-isoindole-1,3(2H)-dione (**C4**)

Yield: 85%. Yellow crystals: mp 210–212 °C (CHCl_3_/hexane); **GC/MS** (70 eV) m/z (%): 308 (M^+^, 100); **IR** (KBr): 1787, 1723 (C=O) cm^–1^; **^1^H NMR** (500 MHz, DMSO-*d*_6_): *δ* 2.45 (s, 3H, C*H*_3_), 2.52 (s, 3H,C*H*_3_) 46 7.35 (d, *J =* 8.1 Hz, 1H, Ar), 7.75 (d, *J* = 7.8 Hz, 1H, Ar), 7.85–7.94 (m, 4H, Ar); **^13^C NMR** (125 MHz, DMSO-*d*_6_): *δ* 21.6 (1C), 21.9 (1C), 121.9 (1C), 122.4 (1C), 124.5 (1C), 124.9 (1C), 128.4 (1C), 128.9 (1C), 129.7 (1C), 131.8 (1C), 133.3 (1C), 135.4 (1C), 136.4 (1C), 147.1 (1C), 147.5 (1C), 151.6 (1C), 165.0 (1C). Anal. calcd for C_17_H_12_N_2_O_2_S 0.25H_2_O (312.85): C 65.26; H 4.03; N 8.95. Found: C 65.66; H 3.95; N 8.99.
